# Primary subacute hematogenous osteomyelitis in children: a clearer bacteriological etiology

**DOI:** 10.1007/s11832-016-0739-3

**Published:** 2016-05-12

**Authors:** Vasiliki Spyropoulou, Amira Dhouib Chargui, Laura Merlini, Eleftheria Samara, Raimonda Valaikaite, Georgios Kampouroglou, Dimitri Ceroni

**Affiliations:** Service of Pediatric Orthopedics, Department of Child and Adolescent Medicine, University Hospitals of Geneva, 6, rue Willy Donzé, Geneva 14, 1211 Switzerland; Pediatric Radiologic Unit, University Hospitals of Geneva, Geneva 14, 1211 Switzerland

**Keywords:** Subacute, Osteomyelitis, Etiology, Microbiology, *Kingella kingae*, MSSA

## Abstract

**Background:**

This study aimed to describe the spectrum of pediatric primary subacute hematogenous osteomyelitis (PSAHO) and to investigate its bacterial etiology.

**Methods:**

Sixty-five consecutive cases of PSAHO admitted to our institution over a 16-year period (2000–2015) were retrospectively reviewed to assess their laboratory and radiographic imaging features, as well as their bacteriological etiology.

**Results:**

On evaluation, white blood cell count and C-reactive protein were normal in 53 (81.5 %) and 34 cases (52.3 %), respectively, whereas the erythrocyte sedimentation rate was superior to 20 mm/h in 44 cases (72.1 %). Blood cultures failed to identify the pathogen in all but one patient, and classic bone sample cultures only managed to isolate the pathogen in five cases (11.6 %). Use of polymerase chain reaction (PCR) assays on bone aspirates or blood allowed the causative microorganism to be isolated in a further 22 cases. Using classic cultures and PCR assays together resulted in pathogen detection in 27 cases (62.8 % of the children bacteriologically investigated), with *Kingella kingae* being the most frequently reported microorganism.

**Conclusions:**

Two distinct forms of PSAHO should be distinguished on the basis of age of patients and bacteriological etiology. The infantile form affects children aged between 6 months and 4 years and is predominantly due to *K. kingae*. The juvenile form involves children aged >4 years and *Staphylococcus aureus* appears to be the main bacteriological etiology. Appropriate nucleic amplification assays drastically improve the detection rate of the microorganisms responsible for PSAHO.

Level of evidence: Case series, level IV.

## Introduction

Subacute hematogenous osteomyelitis is an infectious process characterized most of the time by moderate localized bone pain, mild or no systemic manifestations, non-contributory laboratory results, negative blood cultures, but positive radiological findings [1–12]. According to King and Mayo, any osseous infectious process lasting >2 weeks without acute symptomatology can be referred to as subacute osteomyelitis [8]. Subacute hematogenous osteomyelitis is most likely due to an atypical host−pathogen relationship that may comprise any combination of increased host resistance, decreased virulence of the causative organism, and/or prior antibiotic exposure [3, 4, 10, 13, 14]. The primary form of subacute hematogenous osteomyelitis (PSAHO), which occurs mainly in children, must be distinguished from acute osteomyelitis which has been modified by inadequate or partial treatment with antibiotics and from other conditions (such as chronic recurrent multifocal osteomyelitis and SAPHO syndrome—synovitis, acne, pustulosis, hyperostosis and osteitis) [15]. In many cases, cultures fail to identify the causative organism, especially when fine-needle aspiration is performed. Surgical drainage may yield positive cultures in 40–75 % of patients. When positive, *Staphylococcus aureus* is currently considered as the most common organism isolated [5, 13]. Some reports have suggested an increasing incidence of this form of osteomyelitis [7] and a higher prevalence in certain countries [6]. Gledhill described four radiological types found only in the long bones [4]. Roberts et al. expanded and modified the classification into six forms to include the spine and the pelvis, the lesions being classified as metaphyseal, diaphyseal, epiphyseal, vertebral, and pelvic [10]. Unfortunately, this classification lacks such lesions as apophyseal, transphyseal or small-bone lesions. Here, we aimed to describe the spectrum of PSAHO seen in children, to characterize their laboratory findings and to investigate their bacterial etiology.

## Material and methods

After approval by the Children’s Hospital Review Board, the medical charts of all children and adolescents admitted to our institution for PSAHO between January 2000 and December 2015 were retrospectively reviewed. Eligible patients for enrolment in the present study had to sustain an osseous infectious process confirmed by radiological findings lasting >2 weeks without acute symptomatology or few contributory laboratory results. Analyses were based on clinical records, demographics (age and gender), body temperature, the bone involved, and laboratory data including bacterial cultures (from blood and bone samples), white blood cell (WBC) count, platelet count, erythrocyte sedimentation rate (ESR) and serum C-reactive protein (CRP). Blood cultures were analyzed for isolating the microorganism responsible for PSAHO. When realized, the bone aspirate sample was sent to the laboratory for Gram staining and immediate inoculation onto Columbia blood agar (incubated under anaerobic conditions), CDC anaerobe 5 % sheep blood agar (incubated under anaerobic conditions), chocolate agar (incubated in CO_2_-enriched atmosphere), and brain–heart broth. The media were incubated for 10 days. Since 2007, two polymerase chain reaction (PCR) assays were also used for bacterial identification when classical cultures were negative and initial aliquots (100–200 µl) were stored at −80 °C until processing for DNA extraction. A novel real-time PCR (rtPCR) assay targeting the RTX toxin gene was used in this study [16]. The assay is designed to detect two independent gene targets from the *K. kingae* RTX toxin locus, namely genes *rtxA* and *rtxB*. This PCR assay specific to *K. kingae* was used to analyze different biological samples such as bone biopsy specimen or peripheral blood. Since September 2009, we also started realizing oropharyngeal swab PCR for children aged 6 months to 4 years, as we demonstrated that a simple technique of detection of *K. kingae* RTX toxin genes in the oropharynx provides strong evidence that this microorganism is responsible for the osteoarticular infection, or even stronger evidence that it is not [12]. Finally, broad-range PCR amplification of the 16S rRNA gene was performed using primers BAK11w, BAK2, and BAK533r (Eurogentec, Seraing, Belgium). Radiographic studies of the affected anatomical regions included plain radiographs, and magnetic resonance imaging scans. Radiographic studies were interpreted independently by a board-certified pediatric radiologist and by a senior pediatric orthopedist. Three categories of patients were defined—the first group involved only infants aged <6 months (neonates), since this age corresponds to the end of maternally derived immunity. The second group (infantile) comprised children aged between 6 and 48 months, as it is currently recognized that *K. kingae* is the main pathogen responsible for OAI. Finally, the third group (juvenile and adolescents) included children aged >4 years and <16 years. Descriptive analyses (arithmetic mean, standard deviation and range) were used to describe (1) participant characteristics, (2) clinical parameters such as temperature, (3) results of blood tests, and (4) results of bacteriological investigations.

## Results

### Epidemiology and skeletal distribution

This study reviewed the medical charts of 75 patients. Sixty-five children with PASHO were included while 10 presenting clinical characteristics of chronic recurrent osteomyelitis were excluded. The median age (35 female, 30 male) was 38.1 ± 37.4 months (range 9 months to 12 years). No child was aged <6 months, whereas 55 children (84.6 %) were aged <4 years at the onset of infection. PSAHO mainly affected the spine (22 cases), the femur (16 cases) and the tibia (6 cases). The locations of other lesions are summarized in Table 1. The infection sites were located in long bones (33 cases), tarsal bones (7 cases) and flat bones (one case), patella (two cases) with the remaining 22 cases affecting the spine. According to Roberts’ classification (Table 2), there were 19 cases of type Ia-Ib lesions, one type II, 13 type V, and 22 type VI. The remaining ten cases could not be classified according to Roberts’ criteria—seven cases with affected tarsal bones, two with affected patella and one with affected pelvic bone. Only two patients had prior antibiotic exposure (<48 h) and functional impairment was present in all cases. Five children presented fever on admission (rectal temperature >38 °C), and the remaining 60 children were afebrile (92.3 %).

**Table 1:** Bone localization of subacute osteomyelitis

Infection location	Number of cases
Spine	22
Pelvis	1
Femur	16
Tibia	6
Patella	2
Fibula	3
Tarsal bone	7
Humerus	2
Radius	2
Ulna	4

**Table 2:** Radiological classification of subacute osteomyelitis (10)

Type	Description of radiological features
I	Metaphyseal abscesses characterized by: a. Punched out lucency without significant marginal reaction b. Punched out lesions with sclerotic margin (Brodie’s abscess) Either of these lesions may involve or cross the epiphyseal plates
II	Eccentrically placed metaphyseal lucency eroding cortex
III	Diaphyseal cortical abscess Sequestrum may occur
IV	Diaphyseal medullary abscess Reactive periosteal new bone formation is common
V	Epiphyseal abscess Usually eccentric in the epiphysis
VI	Vertebral osteomyelitis

### Inflammatory markers (cf Table 3)

When considering appropriate age cut-off values, WBC count was normal (<12,000 cells/µL) in 53 cases (81.5 %), and the mean value was 9,545 cells/µL (range 4,950–15,700 cells/µL). CRP (normal value <10 mg/L) was within normal range in 34 cases (52.3 %), while its mean value was 39.6 ± 28.4 mg/L in the remaining 31 cases. When carried out (61 cases), the ESR was superior to 20 mm/h in 44 cases (72.1 %), with a mean value of 31 ± 14.4 mm/h (range 21–64 mm/h). Thirty-six children (55.4 %) had abnormal platelet counts, with a mean value of 411,800 ± 117,235 platelets/µL (range 212,000–700,000 platelets/µL). When all data were available (61 cases), blood inflammatory markers (WBC, ESR and CRP) were all within normal ranges in 12 cases, abnormal for one marker in 24 children, and abnormal for two in 25 children.

**Table 3:** Blood examinations and bacteriological investigation results according to patient age

	<4 years	>4 years
No. of patients	55	10
Mean age (months)	24.2 ± 9.5	114.4 ± 41.8
WBC count (cells/µL)	9.819 ± 2.854	9.050 ± 1.787
Cases with CRP > 10 mg/L	26/55	5/10
Cases with ESR > 20 mm/h	41/53	3/8
Positive blood cultures	0/50	1/10
Positive bone samples cultures	1/33	4/9
Positive rtPCR *Kingella kingae* (blood or bone samples)	20/21	2/2
Microorganisms isolated	*Kingella kingae*, 20 *Mycobacterium tuberculosis*, 1	MSSA, 4 *Kingella kingae*, 2

### Bacteriological investigations (cf Table 3)

Blood cultures were obtained for 60 children, even in the absence of pyrexia. A pathogen failed to be identified from blood cultures in all but one patient (1.7 %), where it was positive for Methicillin-susceptible *Staphylococcus aureus* (MSSA). Open or percutaneous surgical drainage and aerobe/anaerobe bacterial cultures of bone material were carried out for 43 patients. Twenty children with spondylodiscitis or vertebral osteomyelitis, and two with subacute hematogenous osteomyelitis of the pelvis and greater trochanter, received neither open nor percutaneous surgical drainage. In five cases (11.6 % of all bone samples cultures), the pathogen was isolated from bone samples using classic cultures (four MSSA and one *Mycobacterium tuberculosis*). Use of PCR assays during the 2007–2015 period allowed the causative microorganism to be isolated in 22 cases (bone aspirates positive for *K. kingae*,19 cases; blood positive for *K. kingae*, two cases; bone aspirate and blood positive for *K. kingae*, one case). Considering the results from classic cultures and PCR assays together, a pathogen was thus detected in 27 cases (62.8 % of children investigated bacteriologically). Until the end of 2006, bacteriological investigations allowed identification of the pathogen in 12.5 % of cases (2/16), whereas since 2007, 25 responsible microorganisms were detected in 29 children (86.2 %). PCR assays were performed in 26 children, and were positive in 22 cases (84.6 %). Thirty-two children aged between 6 months and 4 years sustained oropharyngeal swab rtPCR specific for *K. kingae*, and these investigations were positive in 31 cases, providing strong evidence that this microorganism was responsible for the osteoarticular infection.

### Spondylodiscitis

During the study period, 22 children (9 female, 13 male) with spondylodiscitis or vertebral osteomyelitis met the clinical and radiological criteria for the study. The mean age of the children was 35.3 ± 39.1 months (range 11 months to 12.5 years). None of them had a rectal temperature >38 °C at admission, and the mean temperature was 37 °C. Blood cultures identified a pathogen in one patient who was positive for MSSA (12.5-year-old patient). When carried out, *K. kingae*-specific PCR assays on throat swabs were positive in all cases (13/13), and of these, three were positive in peripheral blood (3/11). In three patients who underwent percutaneous surgical drainage, the pathogen could be isolated from classic cultures of bone/disk samples in one case (MSSA). In another case, the pathogen was positive on a bone/disk sample using PCR assay (*K. kingae*), but failed to be identified by classic aerobe/anaerobe cultures. None of the 22 children underwent surgical treatment, and all sustained complete recovery with only antibiotics.

## Discussion

Subacute hematogenous osteomyelitis is a distinct form of osteomyelitis difficult to diagnose because the disease has an insidious onset, mild symptoms, lacks a systemic reaction and supportive laboratory data are often inconsistent. Thus, accurate diagnosis is usually delayed until after a lytic bone lesion has occurred and has come into view on plain radiographs. The clinical course of subacute osteomyelitis is most likely the result of an atypical host−pathogen relationship that may comprise any combination of increased host resistance, decreased virulence of the causative organism and/or prior antibiotic exposure [3, 4, 10, 13, 14]. *S aureus* is considered to be the most common causative organism, but other organisms may be encountered *(Streptococcus*, *Pseudomonas*, *Haemophilus influenzae* and coagulase-negative *S. aureus)*.

Two main clinical forms seem to emerge from this case series on pediatric PSAHO which seem to differ depending on patient age and bacteriological etiology. The first form (the infantile form) affects children aged between 6 months and 4 years. Approximately 85 % of all PSAHO affects patients in this age group, and *K. kingae* is the only microorganism cultured in our series. In these particular situations, the clinical course of PSAHO is most likely explained by the naturally low virulence of *K. kingae*. In fact, a *K. kingae* osteoarticular infection (OAI) is characterized by a mild-to-moderate clinical and biological inflammatory response, whilst having few, if any, criteria evocative of OAI [17–22]. Thus, for many children in this age group, diagnosis of OAI is usually made late, conforming to the definition of PSAHO. The second form (the juvenile form) affects children aged >4 years and MSSA becomes the main bacteriological etiology of PSAHO. In this situation, PSAHO is most likely the result of an increased host resistance, and we can hypothesize that the children who develop this resistance against MSSA become able to contain the bone infection. On this subject, MSSA colonization is recognized as being more frequent among younger children [23]. Remarkably, 20 % of individuals are persistently colonized in the nose and 30 % are transiently colonized [24]. Although colonization predisposes an individual to MSSA infection, colonized individuals may experience MSSA infection less severely than non-colonized individuals [25]. This raises the question of whether colonization could induce low-level adaptive immunity so that subsequent infections become milder [24].

The results of the present study also confirm that laboratory data often do not help the diagnosis of PSAHO. Blood tests generally show an increased ESR (72.1 % of cases), whereas WBC count and CRP are likely to be normal or perhaps only slightly elevated. In fact, abnormal WBC counts were recorded in only 18.5 % of cases in this series and abnormal CRP in 47.7 %. The lack of a significant systemic response may simply reflect an adequate local host response to a pathogen of low virulence, such as *K. kingae*, or the presence of low-level adaptive immunity so that subsequent infections become milder. All the blood cultures in this series were negative, except one positive for MSSA. When performed, 38 bone aspirates failed to isolate the causative pathogen on classic cultures in 88.4 % of the cases. Above all, the present study suggests that appropriate nucleic amplification assays drastically improve the detection rate of pathogens responsible for PSAHO, and the rtPCR assay specific to *K. kingae* should be routinely used in young children with joint or bone complaints. In fact, using the rtPCR assay specific for *K. kingae* in children aged <4 years seems to improve isolation of the causative pathogen since the organism using this regimen was recognized in 22 cases.

More than 90 % of proven PSAHO due to *K. kingae* (20 cases) occurred in children aged <4 years, but two children aged >4 years inexpectantly presented PSAHO due to this microorganism. *S. aureus* was nevertheless responsible for most of the PSAHO (57.1 % %) in children aged >4 years. The high rate of sterile blood cultures and the frequent failure to identify the causative pathogen, even on surgical biopsy specimens, constitute another key point very suggestive that a large proportion of infantile-form PSAHO cases are probably due to fastidious microorganisms of low-virulence such as *K. kingae*.

PSAHO in children follows a benign course and the recommended treatment for subacute osteomyelitis with radiographic evidence of lucent lesions or nidus is currently curettage, biopsy and culture followed by antibiotics [17, 18, 26, 27]. Many authors even suggest that antibiotics alone may be adequate and that surgery should be considered only for ‘aggressive lesions’ and those which do not respond to antibiotics [5, 6]. It is, however, generally agreed that treatment should not be initiated until proper drainage and bacteriological samples have been obtained [17, 18, 26, 27]. In children aged <4 years, antibiotherapy should be above all directed against *K. kingae*, whereas the antibiotic regimen should focus on MSSA for children aged >4 years, as this microorganism is the bacteria most often associated with PSAHO.

## Conclusion

PSAHO is a distinct form of osteomyelitis characterized by insidious onset, mild symptoms and few, if any, systemic reactions and anomalies in laboratory data. Two distinct forms seem to be distinguished on the basis of patient age and bacteriological etiology.

The infantile form affects children aged between 6 months and 4 years, and most of the time it is due to *K. kingae*. The juvenile form involves children aged >4 years, and *S. aureus* appears as the main bacteriological etiology. Although blood and bone cultures usually fail to isolate the causative pathogen, appropriate nucleic amplification assays significantly improve the detection rate of microorganisms responsible for PSAHO.
